# The Correlation between Retinal and Choroidal Thickness with Age-Related White Matter Hyperintensities in Progressive Supranuclear Palsy

**DOI:** 10.3390/jcm12206671

**Published:** 2023-10-22

**Authors:** Maddalena De Bernardo, Francesco Diana, Marco Gioia, Martina De Luca, Maria Francesca Tepedino, Maria Teresa Pellecchia, Nicola Rosa, Paolo Barone, Marina Picillo

**Affiliations:** 1 Eye Unit, Department of Medicine, Surgery and Dentistry “Scuola Medica Salernitana”, University of Salerno, 84084 Fisciano, Italy; mdebernardo@unisa.it (M.D.B.); martinadelux@gmail.com (M.D.L.); nrosa@unisa.it (N.R.); 2 Neuroradiology Unit, University Hospital San Giovanni di Dio e Ruggi d’Aragona, 84131 Salerno, Italy; fradiana@unisa.it; 3 Interventional Neurology Department, Vall d’Hebron University Hospital, 08035 Barcelona, Spain; 4 Center for Neurodegenerative Diseases (CEMAND), Department of Medicine, Surgery and Dentistry “Scuola Medica Salernitana”, University of Salerno, 84084 Fisciano, Italy; mftepedino@unisa.it (M.F.T.); mpellecchia@unisa.it (M.T.P.); pbarone@unisa.it (P.B.); mpicillo@unisa.it (M.P.)

**Keywords:** progressive supranuclear palsy, retina, choroid, optical coherence tomography, neuroradiology, white matter age-related changes

## Abstract

Progressive supranuclear palsy (PSP) is a rare neurodegenerative disease. Recently, several retinal layers in PSP compared to healthy controls. were found to be thinner. However, no studies evaluating the correlation between retinal layers and cerebral white matter changes, nor eventual choroidal changes in PSP, have been conducted so far. The goals of the present study were to explore potential differences in choroidal structure between PSP and healthy controls, and to describe the relationship between retinal layers’ thickness and volume, using spectral-domain optical coherence tomography (SD-OCT) and age-related white matter change scores (ARWMC) using magnetic resonance imaging (MRI) of the brain. Choroidal structures of 26 PSP patients and 26 healthy controls using standard SD-OCT with an enhanced depth imaging (EDI) approach were analyzed; then, retinal the structures of 16 of these PSP patients using standard SD-OCT were examined; finally, the same patients underwent brain MRI, and their cerebral white matter changes were calculated. Non-statistically significant differences between PSP patients’ and healthy controls’ choroidal structure were found. On the contrary, PSP patients’ inner retinal layers (INR), retinal pigmented epithelium (RPE) and all retinal layers’ thicknesses in the macular region were found to be significantly correlated with ARWMC, independently from age and axial length (AL). PSP patients’ neurological alterations go hand in hand with retinal ones, independently from age and axial length. Our results suggest a mutual relationship between cerebral and retinal structure pathological alterations. On the other hand, no significant differences in the choroidal evaluation compared to healthy controls have been found.

## 1. Introduction

Progressive supranuclear palsy (PSP) is a rare, degenerative disease of the central nervous system. It is associated with a specific four-repeat tau neuropathology and consists of dementia, balance impairment, akinesia and conjugate gaze palsy in a vertical direction [1,2]. A growing body of evidence suggests an important role and contribution of vascular pathology to a variety of neurodegenerative diseases, including PSP. Notably, cerebral small vessel disease (CSVD) is considered the main responsible cause of Alzheimer’s disease as well as PSP [3,4].

Many neurodegenerative diseases can be studied in vivo and in a non-invasive method through the examination of the eye. In particular, changes in the neuronal and vascular structure of the retina may reflect similar changes occurring in the corresponding brain structures [5].

The retina and choroid are currently examined using spectral-domain optical coherence tomography (SD-OCT), a safe and reproducible imaging technique, offering high-resolution retinal and choroidal scans without and with an enhanced depth imaging (EDI) approach, respectively [6].

On OCT images, choroidal structure is estimated, determining subfoveal, nasal and temporal choroidal thickness (ChT) and scores as total choroidal area (TCA), luminal choroidal area (LCA), stromal choroidal area (SCA) and the choroidal vascularity index (CVI) [7].

Previously, we demonstrated PSP patients to show significant thinning of the inner retinal layer (IRL), the ganglion cell layer (GCL) and the inner and outer plexiform layer (IPL and OPL, respectively) compared to age-matched healthy controls (HC) [8]. To date, there are no studies examining the link between CSVD and retinal layers’ thickness or choroidal structure in patients with PSP.

The purpose of the present study is to describe the potential variations in the choroidal structure in PSP patients compared with HC. In addition, the relationship between CSVD evaluated with brain magnetic resonance imaging (MRI) and both retinal layers’ thickness and volume, as well as choroidal thickness and vascular scores, have been explored [9].

## 2. Materials and Methods

### 2.1. Participants and Methods

The study is consistent with the Tenets of the Declaration of Helsinki and Institutional Review Board (CECS, Cometico Campania Sud prot. n°16544). Approval has been achieved. Written clinical permission was acquired after all participants had been informed on the objective of the study. Between June 2018 and December 2019, a total of 26 PSP patients diagnosed according to the Movement Disorders Society (MDS) criteria and 26 healthy controls with a comparable age and axial length (AL) were included and examined in this prospective, non-randomized study [1]. Patients affected by corneal leukomas, diabetic retinopathy, hypertensive retinopathy from grade II to IV, senile macular degeneration, central serous chorioretinopathy, glaucoma, macular hole, uveitis, hypertension not controlled by medications, autoimmune disorders and ocular or systemic diseases, which could have changed retinal features, have been excluded. One eye of each patient was evaluated.

### 2.2. Clinical and Instrumental Examination

Preoperative visit consisted of a comprehensive ophthalmological examination, including medical history collection, visual acuity measurement using the Snellen chart, both with and without vision correction. Additionally, an evaluation of the anterior segment, intraocular pressure (IOP) measurement, fundus examination, AL measurements with IOLMaster (Carl Zeiss Meditec AG, Jena, Germany, version 5.4.4.0006) and spectral-domain (SD) OCT evaluation using EDI mode in 840 nm (Spectralis; Heidelberg Engineering; Heidelberg, Germany, version 6.0) was conducted.

### 2.3. Imaging Protocol and Image Analysis

A skilled examiner, unaware of the patients’ distribution, obtained a horizontal 30° linear OCT B-scan passing through the fovea. The scanning angle was set at 308 and each B-scan consisted of 100 frames. Both EDI and non-EDI approaches were used, but only EDI-OCT images with a high signal-to-noise ratio (minimum of 20 dB) that allowed for adequate visualization of the choroid were selected for analysis. To minimize bias related to diurnal ChT variations, all examinations were conducted between 2:00 p.m. and 3:00 p.m.

The measurements were performed using the software integrated into the Heidelberg Eye Explorer HEYEX device, version 5.3. An experienced OCT evaluator (MDB) reviewed all collected and analyzed imaging data. The thickness of the retinal layers within a 1 mm diameter circle centered on the fovea, as well as in five foveal and parafoveal zones with a diameter of 3 mm, was determined according to the Early Treatment Diabetic Retinopathy Study grid (ETDRS), following established protocols [8].

The retinal layers’ segmentation, the macular layers’ thickness (Mt) and volume (Mv) in the 1 mm diameter-macular area and their mean thickness (Meant) and volume (Meanv) in the 3 mm diameter-macular area were achieved using the instrument’s automatic algorithm on volume scan without EDI approach.

**Figure 1:**
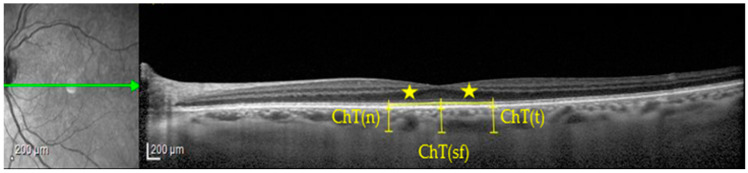
Choroidal thickness (ChT) measurement in a PSP patient. ChT(n), nasal choroidal thickness; ChT(sf), subfoveal choroidal thickness; ChT(t), temporal choroidal thickness; star symbol, 750 microns of horizontal distance from fovea.

Choroidal assessment was obtained on a single line scan with EDI approach: ChT in subfoveal region and at 750 microns nasally and at 750 microns temporally to the fovea were calculated on images in micron scale, using a line perpendicular to retinal pigment epithelium (RPE) that extends between RPE–Bruch interface and sclerochoroidal junction (as shown in Figure 1).

Macular B-scan images were exported using a 1:1 pixel ratio and processed utilizing the ImageJ 1.52q software from the National Institutes of Health in Bethesda, MD, USA. The examiner, who was unaware of the patient’s status, as suggested by previous studies, measured and calculated TCA, LCA, SCA, and CVI [7,8].

**Figure 2:**
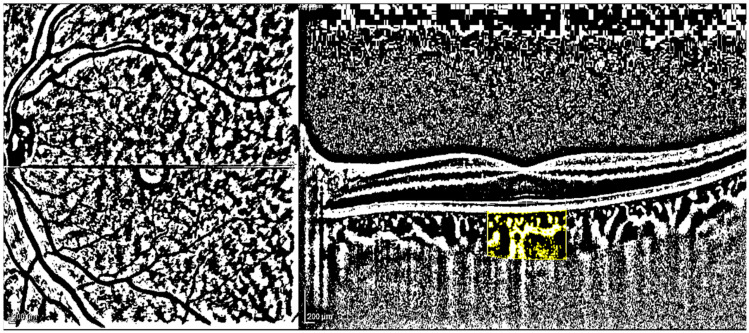
Choroidal vascularity index (CVI) measurement. Legend: yellow polygon: evaluated choroidal area; black areas: choroidal vascular lumens; white areas, choroidal stroma.

To determine the scanned choroidal area in OCT B-scans, the polygon tool was used after converting the image to 8-bit. Niblack’s auto-local threshold was then applied to binarize and identify LCA and SCA areas, as illustrated in Figure 2, following established protocols [10]. Color thresholding highlighted TCA, which was added to the region of interest (ROI) manager. In each eye, the measurements for TCA and LCA were obtained while CVI (defined as LCA/TCA) was computed [11]. Finally, SCA results are derived by subtracting LCA from TCA.

Both PSP patients and healthy controls were evaluated with an IOLMaster (5.4.4.0006; Carl Zeiss Meditec AG, Jena, Germany) to measure the AL. The mean of at least three measurements with the highest signal-to-noise ratio was considered. To obtain comparable data, the assessment of AL of the groups was performed, and no statistically significant difference was shown.

### 2.4. Brain MRI Protocol and Vascular Scoring

Brain MRI was performed for 16 out of 26 PSP patients within a time window of 15 days before or after the eye examination [9]. All patients underwent an MRI scan of the brain at 3 Tesla, with an imaging protocol including axial Fast Spin- Echo T2-weighted and FLAIR sequences.

White matter changes were defined as ill-defined hyperintensities larger than 5 mm on both T2 and FLAIR images [12]. The differentiation of lacunes and perivascular spaces was based on size and signal intensity. We used the validated visual age-related white matter changes scale (ARWMC) to rate white matter changes. The degree of white matter changes was evaluated on a 4-point scale (from the absence of any lesion—score 0, to the presence of confluent lesions—score 3), in five different regions on each hemisphere (frontal, parieto-occipital, temporal, infratentorial and basal ganglia) (total ARWMC score from 0 to 30) [12]. The ARWMC scale was performed by an experienced neuroradiologist (FD), blinded to the results of both clinical and eye evaluation.

### 2.5. Statistical Analysis

To assess the normal distribution of the data, a Kolmogorov–Smirnov test was conducted. The comparison of categorical variables was performed using a chi-squared test. Parametric or non-parametric tests, namely the unpaired samples *t*-test or Mann–Whitney U test, were used to find differences between patients’ and controls’ ChT and vascular scores, namely TCA, LCA, SCA and CVI. Spearman’s correlation was used to explore the link between the ARWMC and (a) retinal layers’ thickness, (b) retinal layers’ volume, (c) choroidal thickness and (d) choroidal vascular scores, namely TCA, LCA, SCA and CVI.

The statistical analyses were conducted using SPSS version 20. A two-sided *p* value of less than 0.05 was chosen as the threshold for determining statistical significance. Since this study had an exploratory focus, no correction for multiple comparisons was implemented.

All measurements were acquired as the distance from the RPE–Bruch interface to the sclerochoroidal junction, drawing a perpendicular line to the retinal pigment epithelium (RPE).

In this area, total choroidal area (TCA), luminal choroidal area (LCA), then stromal choroidal area (SCA) and choroidal vascularity index (CVI) utilizing ImageJ 1.52 were evaluated. After drawing the polygon tool, the image was converted into 8-bit, then Niblack’s auto-local threshold and color threshold were applied.

## 3. Results

Demographic characteristics and axial length of 26 PSP patients and 26 healthy controls are listed in Table 1. The two groups were similar in terms of age and AL. Enrolled PSP patients had a disease duration of (mean ± standard deviation) 2.41 ± 1.08 years.

ChT in subfoveal, nasal and temporal sites and choroidal scores as TCA, LCA, SCA and CVI of both patients and healthy controls are shown in Table 2. No significant difference regarding ChT and choroidal vascularity scores was found between the two groups.

Thickness and volume of retinal layers in macular and five central regions (diameter 3 mm) for PSP patients are shown in Table 3.

Finally, ARWMCs for each lobe of each hemisphere for PSP patients are summarized in Table 4.

In PSP patients, ARWMC score in occipital lobes is positively correlated with mtRPE (r = 0.502, *p* = 0.048) and negatively with macular thickness (Mt) (all layers) (r = −0.533, *p* = 0.034), mtIRL (r = −0.533, *p* = 0.033) and MvIRL thickness (r = −0.499, *p* = 0.049), Meant inner nuclear layer (INL) (r = −0.535, *p* = 0.33) and MeanvINL (r = −0.519, *p* = 0.039). None of these is correlated with age and AL: instead, there is a negative correlation between age and MeantIPL (r = −0.716, *p* = 0.002), MeanvIPL (r = −0.794, *p* = 0.00), MeantGCL (r = −0.617, *p* = 0.011), MeanvGCL (r = −0.672, *p* = 0.004), Meant retinal nerve fiber layer (RNFL) (r = −0.552, *p* = 0.027), and a positive correlation between AL and MeantOPL (r = 0.0529, *p* = 0.035).

No other significant correlations were detected.

## 4. Discussion

Herein, we compared ChT in PSP patients and healthy controls with similar age and axial length, as both such parameters may influence ChT and retinal measurements [13]. According to the literature, age and ChT, especially for the subfoveal one, have a negative correlation [14,15]. Similarly, ChT indirectly varies with AL [13]. According to Gyawali P et al. ChT decreases by 40.32 μm (95% CI: 32.58 μm to 48.06 μm, and *p* < 0.001) for every 1 mm increase in the axial length [16].

Although we failed to detect significant changes in ChT in PSP compared to healthy controls (*p* > 0.05) (Supplemental Figure S1) and a significant correlation between ChT and ARWMC in PSP (ρ_s_ > 0.05), we demonstrated specific correlations between retinal layers and ARWMC in PSP.

In addition to a growing body of evidence [17,18,19], we previously demonstrated that PSP presents significant differences in thickness of specific retinal layers compared to HC, with similar age and AL. In particular, a significant thinning of the IRL, OPL, IPL and GCL was detected in PSP patients compared to HC, when considering the central five macular regions (as showed in Supplemental Figures S2 and S3) [8].

ChT and structures have been studied in several diseases [10,20,21], but the possible involvement of choroidal structure in retinal diseases is controversial [22,23].

Characterizations of retinal and choroidal microvasculature were conducted in Parkinson’s disease, but no data are available in PSP [24,25].

Cerebral small vessel disease (CSVD), which can be assessed with the ARWMC, is considered a major pathogenic contributor to several neurodegenerative diseases, such as Alzheimer’s disease and PSP [3,4,12,26].

In the present study, the presence of an association between PSP patients’ neuroradiological and ophthalmologic degenerations is the innovative finding. The negative correlation of macular retinal layers’ thickness, especially the INL, and the positive correlation of RPE of PSP patients with the ARWMC score in their occipital lobes further support the mutual relationship between retinal and occipital lobe structures. In line with previous reports, greater retinal thinning may represent a marker of a more severe form of disease involving the occipital lobe [8,27].

The association between RPE and ARWMC is subject to debate. RPE arises from the neuroectoderm, a layer of cells that develop into optic grooves. These grooves then form optic vesicles under the influence of mesenchymal stimulation. The optic vesicle further develops by invaginating at four weeks gestation, resulting in the formation of an optic cup with two layers: an internal layer and an external one responsible for generating RPE [28]. Similarly, cerebral white matter consists of myelinated axon tracts that facilitate communication between different brain regions; this white matter also originates from the neuroectodermal germ layer, just like its covering myelin [29,30].

In contrast, the choroidal vascular network arises from the mesoderm and is initially part of the uveal vascular system that surrounds the outer layer of the optic cup [31]. By studying embryology and examining similarities between cerebral and retinal microvasculatures’ composition [32], it is possible to hypothesize about a significant correlation between findings in both areas.

No correlation between white matter changes and choroid of PSP patients was found, but further studies are needed. The potential influence of the choroid on retinal development and pathology should not be disregarded, given the role of mesenchymal stimulus in neuroectodermal differentiation [28] and evidence suggesting similarities between embryology, anatomy and metabolism among retinal, choroidal and cerebral vascularization. Geerling CF et al. conducted a study comparing 20 patients diagnosed with cerebral small vessel disease (CSVD) to 10 healthy controls. They found significant differences in choriocapillaris reflectivity standard deviation measured by optical coherence tomography angiography (OCT-A) between the cases and controls (*p* = 0.039). Additionally, they observed associations between logarithmically transformed white matter index values and characteristic lesions seen on MR images with vessel density, vessel diameter index in superficial and deep plexus layers as well as choriocapillaris parameters during unadjusted analyses [33].

Furthermore, correlations are independent from age and AL, factors that could introduce a bias in the analysis of correlation [34]. In terms of ChT, we failed to detect any difference between the two groups.

We acknowledge some limitations of the present study: there is a different gender distribution among the two groups; however, its role in influencing ChT is uncertain [35,36,37,38,39,40,41]. In the HC group, no MRI and ARWMC computation was performed; therefore, the eventual correlation between this neuroradiological score and retinal layers was not evaluated. However, PSP was the main target population of our study. Further ophthalmological investigations together with neuroradiological findings may provide new insights into the physiopathological mechanisms of PSP.

## Figures and Tables

**Table 1:** Demographic characteristics and axial length of PSP patients and HC.

		Total PSP Patients	HC	*p*
*n.*		26	26	0.043
*M/W*		13/13	6/20	
*Age*	*Mean ± SD*	69.89 ± 6.71	71.39 ± 7.56	0.284
	*Median*	72.25	73.91	
	*min–max*	53.08–82.25	48.17–83.25	
*AL*	*Mean ± SD*	23.29 ± 0.82	23.27 ± 0.83	0.923
	*Median*	23.41	23.36	
	*min–max*	22.03–25.34	22.06–25.43	

Abbreviations: PSP, progressive supranuclear palsy; HC, healthy controls; M, men; W, women; SD, standard deviation.

**Table 2:** Comparisons of choroidal vascularization scores between PSP patients and HC.

		PSP Patients (26)	HC (26)	*p*
*ChT subfoveal* (µm)	Mean ± SD	243.15 ± 70	214.58 ± 50.26	0.097
	Range	123–416	125–339	
*ChT nasal* (µm)	Mean ± SD	230.77 ± 70.71	199.38 ± 58.78	0.088
	Range	121–403	99–327	
*ChT temporal* (µm)	Mean ± SD	238.12 ± 67.73	207.19 ± 53.12	0.073
	Range	124–372	130–334	
*TCA* (mm^2^)	Mean ± SD	2.04 ± 0.51	1.86 ± 0.46	0.200
	Range	1.34–3.19	1.12–2.66	
*LCA* (mm^2^)	Mean ± SD	1.36 ± 0.33	1.23 ± 0.31	0.145
	Range	0.91–2.06	0.69–1.78	
*SCA* (mm^2^)	Mean ± SD	0.67 ± 0.21	0.63 ± 0.16	0.621
	Range	0.35–1.13	0.39–0.94	
*CVI*	Mean ± SD	67.3% ± 5%	66.1% ± 3.3%	0.308
	Range	50.1–75.6%	60%–73.1%	

Abbreviations: PSP, progressive supranuclear palsy; HC, healthy controls; M, men; W, women; SD, standard deviation; ChT, choroidal thickness; TCA, total choroidal area; LCA, luminal choroidal area; SCA, stromal choroidal area; CVI, choroidal vascularity index.

**Table 3:** Retinal layers’ thickness in PSP patients.

	PSP Patients (16)		
	Fovea (1 mm Diameter)	Mean ± SD	Range
**Thickness (Mt) (μm)**	*Photoreceptors (Bruch-OLM)*	87 ± 5.45	79–98
	*IRL (OLM-ILM)*	182.19 ± 26.74	148–236
	*RPE (Bruch-RPE)*	15 ± 1.63	12–17
	*ONL*	87.69 ± 10.98	67–108
	*OPL*	24.19 ± 6.66	14–35
	*ONL/OPL*	3.96 ± 1.42	1.97–7.71
	*All layers*	269.19 ± 25.4	231–321
**Volume (Mv) (μm^2^)**	*Photoreceptors (median)*	0.07	0.06–0.08
	*IRL thickness*	0.14 ± 0.022	0.12–0.19
	*RPE*	0.01	
	*ONL (median)*	0.07	0.05–0.08
	*OPL (median)*	0.02	0.01–0.03
	*Macular volume (median)*	0.21	0.18–0.25
	**5 central regions (3 mm)**	**Mean ± SD**	**Range**
**Thickness (Meant) (μm)**	*Photoreceptors*	82.71 ± 3.65	76.2–89.8
	*IRL (median)*	225.50	205.6–264.8
	*RPE*	14.53 ± 1.34	12.8–17.4
	*ONL*	72.25 ± 7.71	52.4–85.6
	*OPL*	30.88 ± 3.42	25.6–36.2
	*ONL/OPL*	2.38 ± 0.40	1.45–2.81
	*INL*	37.05 ± 3.80	31–46
	*IPL*	33.25 ± 4.30	25–45.6
	*GCL*	37.63 ± 6.24	23–45.6
	*RNFL*	19.03 ± 2.09	16.4–25
	*Macular thickness*	312.34 ± 13.91	285.6–343.2
**Volume (Meanv) (μm^2^)**	*Photoreceptors (median)*	0.12	0.108–0.126
	*IRL (median)*	0.33	0.3–0.378
	*RPE (mean ± SD)*	0.02 ± 0.002	0.018–0.024
	*ONL (mean ± SD)*	0.10 ± 0.011	0.122–0.072
	*OPL (median)*	0.04	0.054–0.036
	*INL (median)*	0.06	0.064–0.044
	*IPL (median)*	0.05	0.036–0.178
	*GCL (median)*	0.06	0.036–0.066
	*RNFL (median)*	0.03	0.024–0.038
	*Macular volume (median)*	0.45	0.414–0.49

Abbreviations: PSP, progressive supranuclear palsy; Mt, retinal layer thickness in macular site; Mv, retinal layer volume in macular site; Meant, mean retinal layer thickness in macular site; Meanv, mean retinal layer volume in macular site; IRL: inner retinal layer; INL: inner nuclear layer; IPL: inner plexiform layer; OPL: outer plexiform layer; GCL: ganglion cell layer; RNFL: retinal nerve fiber layer.

**Table 4:** ARWMC scale for PSP patients (*n* = 16).

ARWMC Scale			
	Mean ± SD	Median	Range
**Frontal**	0.5 ± 0.65	0	0–2
**Parietal**	0.16 ± 0.38	0	0–1
**Temporal**	0.06 ± 0.25	0	0–1
**Occipital**	0.13 ± 0.34	0	0–1
**Basal ganglia**	0		
**Total**	1.81 ± 2.56	0	0–8

Abbreviations: PSP. progressive supranuclear palsy; ARWMC. age-related white matter changes.

## Data Availability

Data is unavailable due to privacy or ethical restriction.

## Supplementary Materials

The following supporting information can be downloaded at: https://www.mdpi.com/article/10.3390/jcm12206671/s1, Figure S1: Choroidal thickness (ChT) measurement in a healthy control (HC); Figure S2: Retinal layers’ (GCL, IPL, OPL, IRL respectively) thickness measurements in 5 central macular regions: a PSP patient; Figure S3: Retinal layers’ (GCL, IPL, OPL, IRL respectively) thickness measurements in 5 central macular regions: an HC patient.

## Abbreviations

PSP: progressive supranuclear palsy; OCT: optical coherence tomography; MRI: magnetic resonance imaging; ChT: choroidal thickness; IRL: inner retinal layer; INL: inner nuclear layer; IPL: inner plexiform layer; OPL: outer plexiform layer; GCL: ganglion cell layer; RNFL: retinal nerve fiber layer; ARWMC: age-related white matter changes; EDI: enhanced depth imaging; AL: axial length; TCA: total choroidal area; LCA: luminal choroidal area; SCA: stromal choroidal area; CVI: choroidal vascularity index; mtIRLthickness: IRL thickness in macular zone; vIRLthickness: IRL volume in macular zone; mtRPE: RPE thickness in macular zone; MeantINL: INL mean thickness in 3 mm central macular area; MeanvINL: mean volume in 3 mm central macular area; mtMACULAR THICKNESS all layers: all retinal layers’ total thickness in macular area; MeantIPL: IPL mean thickness in 3 mm central macular area; MeanvIPL: IPL mean volume in 3 mm central macular area; MeantGCL: GCL mean thickness in 3 mm central macular area; MeanvGCL: GCL mean volume in 3 mm central macular area; MeantRNFL: RNFL mean thickness in 3 mm central macular area; MeantOPL: OPL mean thickness in 3 mm central macular area.

## References

[1] Höglinger G.U., Respondek G., Stamelou M., Kurz C., Josephs K.A., Lang A.E., Mollenhauer B., Müller U., Nilsson C., Whitwell J.L. (2017). Clinical diagnosis of progressive supranuclear palsy: The movement disorder society criteria. Mov. Disord..

[2] Boxer A.L., Yu J.-T., Golbe L.I., Litvan I., Lang A.E., Höglinger G.U. (2017). New diagnostics and therapeutics for progressive supranuclear palsy. Lancet Neurol..

[3] Wardlaw J.M., Smith E.E., Biessels G.J., Cordonnier C., Fazekas F., Frayne R., Lindley R.I., O’Brien J.T., Barkhof F., Benavente O.R. (2013). Neuroimaging standards for research into small vessel disease and its contribution to ageing and neurodegeneration. Lancet Neurol..

[4] Lukic M.J., Kurz C., Respondek G., Grau-Rivera O., Compta Y., Gelpi E., Troakes C., van Swieten J.C., Barcelona Brain Bank Collaborative Group, The MDS-Endorsed PSP Study Group (2020). Copathology in Progressive Supranuclear Palsy: Does It Matter?. Mov. Disord..

[5] Cheung C.Y., Chan V.T., Mok V.C., Chen C., Wong T.Y. (2019). Potential retinal biomarkers for dementia: What is new?. Curr. Opin. Neurol..

[6] Stemplewitz B., Kromer R., Vettorazzi E., Hidding U., Frings A., Buhmann C. (2017). Retinal degeneration in progressive supranuclear palsy measured by optical coherence tomography and scanning laser polarimetry. Sci. Rep..

[7] Agrawal R., Ding J., Sen P., Rousselot A., Chan A., Nivison-Smith L., Wei X., Mahajan S., Kim R., Mishra C. (2020). Exploring choroidal angioarchitecture in health and disease using choroidal vascularity index. Prog. Retin. Eye Res..

[8] Picillo M., Salerno G., Tepedino M.F., Abate F., Cuoco S., Gioia M., Coppola A., Erro R., Pellecchia M.T., Rosa N. (2022). Retinal thinning in progressive supranuclear palsy: Differences with healthy controls and correlation with clinical variables. Neurol. Sci..

[9] Picillo M., Tepedino M.F., Abate F., Erro R., Ponticorvo S., Tartaglione S., Volpe G., Frosini D., Cecchi P., Cosottini M. (2020). Midbrain MRI assessments in progressive supranuclear palsy subtypes. J. Neurol. Neurosurg. Psychiatry.

[10] De Bernardo M., Vitiello L., Battipaglia M., Mascolo F., Iovino C., Capasso L., Ciacci C., Rosa N. (2021). Choroidal structural evaluation in celiac disease. Sci. Rep..

[11] Sonoda S., Sakamoto T., Yamashita T., Uchino E., Kawano H., Yoshihara N., Terasaki H., Shirasawa M., Tomita M., Ishibashi T. (2015). Luminal and Stromal Areas of Choroid Determined by Binarization Method of Optical Coherence Tomographic Images. Am. J. Ophthalmol..

[12] Wahlund L.O., Barkhof F., Fazekas F., Bronge L., Augustin M., Sjögren M., Wallin A., Ader H., Leys D., Pantoni L. (2001). A New Rating Scale for Age-Related White Matter Changes Applicable to MRI and CT. Stroke.

[13] De Bernardo M., Cione F., Capasso L., Coppola A., Rosa N. (2022). A formula to improve the reliability of optical axial length measurement in IOL power calculation. Sci. Rep..

[14] Liu B., Wang Y., Li T., Lin Y., Ma W., Chen X., Lyu C., Li Y., Lu L. (2018). Correlation of subfoveal choroidal thickness with axial length, refractive error, and age in adult highly myopic eyes. BMC Ophthalmol..

[15] Zhou H., Dai Y., Shi Y., Russell J.F., Lyu C., Noorikolouri J., Feuer W.J., Chu Z., Zhang Q., de Sisternes L. (2020). Age-Related Changes in Choroidal Thickness and the Volume of Vessels and Stroma Using Swept-Source OCT and Fully Automated Algorithms. Ophthalmol. Retin..

[16] Gyawali P., Jnawali A., Kharal A., Subedi M., Kandel S., Puri P.R., Paudel N. (2023). SubFoveal Choroidal Imaging in High Myopic Nepalese Cohort. J. Ophthalmol..

[17] Albrecht P., Müller A.-K., Südmeyer M., Ferrea S., Ringelstein M., Cohn E., Aktas O., Dietlein T., Lappas A., Foerster A. (2012). Optical Coherence Tomography in Parkinsonian Syndromes. PLoS ONE.

[18] Schneider M., Müller H.-P., Lauda F., Tumani H., Ludolph A.C., Kassubek J., Pinkhardt E.H. (2014). Retinal single-layer analysis in Parkinsonian syndromes: An optical coherence tomography study. J. Neural Transm..

[19] Woo K.A., Shin J.Y., Kim H., Ahn J., Jeon B., Lee J.-Y. (2022). Peripapillary retinal nerve fiber layer thinning in patients with progressive supranuclear palsy. J. Neurol..

[20] De Bernardo M., Altieri V., Coppola A., Gioia M., Rosa N. (2020). Choroidal evaluation in patients under alpha-lytic therapy. Graefe’s Arch. Clin. Exp. Ophthalmol..

[21] Vitiello L., De Bernardo M., Erra L., Della Rocca F., Rosa N., Ciacci C. (2022). Optical Coherence Tomography Analysis of Retinal Layers in Celiac Disease. J. Clin. Med..

[22] De Bernardo M., Salerno G., Gioia M., Capasso L., Russillo M.C., Picillo M., Erro R., Amboni M., Barone P., Rosa N. (2021). Intraocular pressure and choroidal thickness postural changes in multiple system atrophy and Parkinson’s disease. Sci. Rep..

[23] Sevim D.G., Unlu M., Gultekin M., Karaca C., Mirza M., Mirza G.E. (2018). Evaluation of Retinal Changes in Progressive Supranuclear Palsy and Parkinson Disease. J. Neuro-Ophthalmol..

[24] Kamata Y., Hara N., Satou T., Niida T., Mukuno K. (2022). Investigation of the pathophysiology of the retina and choroid in Parkinson’s disease by optical coherence tomography. Int. Ophthalmol..

[25] Robbins C.B., Thompson A.C., Bhullar P.K., Koo H.Y., Agrawal R., Soundararajan S., Yoon S.P., Polascik B.W., Scott B.L., Grewal D.S. (2021). Characterization of Retinal Microvascular and Choroidal Structural Changes in Parkinson Disease. JAMA Ophthalmol.

[26] Nguyen T.-T., Cheng J.-S., Chen Y.-L., Lin Y.-C., Tsai C.-C., Lu C.-S., Weng Y.-H., Wu Y.-M., Hoang N.-T., Wang J.-J. (2021). Fixel-Based Analysis of White Matter Degeneration in Patients with Progressive Supranuclear Palsy or Multiple System Atrophy, as Compared to Parkinson’s Disease. Front. Aging Neurosci..

[27] Armstrong R., Kergoat H. (2015). Oculo-visual changes and clinical considerations affecting older patients with dementia. Ophthalmic Physiol. Opt..

[28] Fuhrmann S. (2010). Eye morphogenesis and patterning of the optic vesicle. Curr. Top. Dev. Biol..

[29] Blumenfeld H. (2010). Areas of the CNS made up mainly of myelinated axons are called white matter. Neuroanatomy through Clinical Cases.

[30] Elshazzly M., Lopez M.J., Reddy V., Caban O. (2023). Embryology, Central Nervous System.

[31] Saint-Geniez M., D’Amore P.A. (2004). Development and pathology of the hyaloid, choroidal and retinal vasculature. Int. J. Dev. Biol..

[32] Patton N., Aslam T., MacGillivray T., Pattie A., Deary I.J., Dhillon B. (2005). Retinal vascular image analysis as a potential screening tool for cerebrovascular disease: A rationale based on homology between cerebral and retinal microvasculatures. J. Anat..

[33] Geerling C.F., Terheyden J.H., Langner S.M., Kindler C., Keil V.C., Turski C.A., Turski G.N., Wintergerst M.W.M., Petzold G.C., Finger R.P. (2022). Changes of the retinal and choroidal vasculature in cerebral small vessel disease. Sci. Rep..

[34] La Marca A., Biondino D., Gioia M. (2022). Comment on Naranjo-Bonilla et al. Retinal and Choroidal Effects of Continuous Positive Airway Pressure as Treatment for Sleep Apnea: Results at 12 Months. Int. J. Environ. Res. Public Health.

[35] Li X.Q., Larsen M., Munch I.C. (2011). Subfoveal Choroidal Thickness in Relation to Sex and Axial Length in 93 Danish University Students. Investig. Opthalmology Vis. Sci..

[36] Park K.-A., Oh S.Y. (2013). Choroidal Thickness in Healthy Children. Retina.

[37] Bidaut-Garnier M., Schwartz C., Puyraveau M.M., Montard M., Delbosc B., Saleh M. (2014). Choroidal thickness measurement in children using optical coherence tomography. Retina.

[38] Mirzania D., Thompson A.C., Robbins C.B., Soundararajan S., Lee J.M., Agrawal R., Liu A.J., Johnson K.G., Grewal D.S., Fekrat S. (2021). Retinal and Choroidal Changes in Men Compared with Women with Alzheimer’s Disease: A Case-Control Study. Ophthalmol. Sci..

[39] Mori Y., Miyake M., Hosoda Y., Uji A., Nakano E., Takahashi A., Muraoka Y., Miyata M., Tamura H., Ooto S. (2021). Distribution of Choroidal Thickness and Choroidal Vessel Dilation in Healthy Japanese Individuals: The Nagahama Study. Ophthalmol. Sci..

[40] Mihara N., Sonoda S., Terasaki H., Shiihara H., Sakono T., Funatsu R., Sakamoto T. (2023). Sex- and Age-Dependent Wide-Field Choroidal Thickness Differences in Healthy Eyes. J. Clin. Med..

[41] Wang L., Wang W., Zhou Z., Wang H., Chakravarthy U., Peto T., Casalino G., Wang K., Li S. (2023). Quantitative Assessment of Choroidal Thickness and Choroidal Vascular Features in Healthy Eyes Based on Image Binarization of EDI-OCT: A Single-Center Cross-Sectional Analysis in Chinese Population. J. Clin. Med..

