# Supplementary material for: The Correlation between Retinal and Choroidal Thickness with Age-Related White Matter Hyperintensities in Progressive Supranuclear Palsy
Source: J Clin Med. 2023 Oct 22;12(20):6671. doi: 10.3390/jcm12206671 (PMC10607459; doi:10.3390/jcm12206671)
Supplement: Supplementary file 1 [file jcm-12-06671-s001.zip › jcm-2639026-supplementary.pdf]

Supplementary Materials:

Figure S1. Choroidal thickness (ChT) measurement in a healthy control (HC).

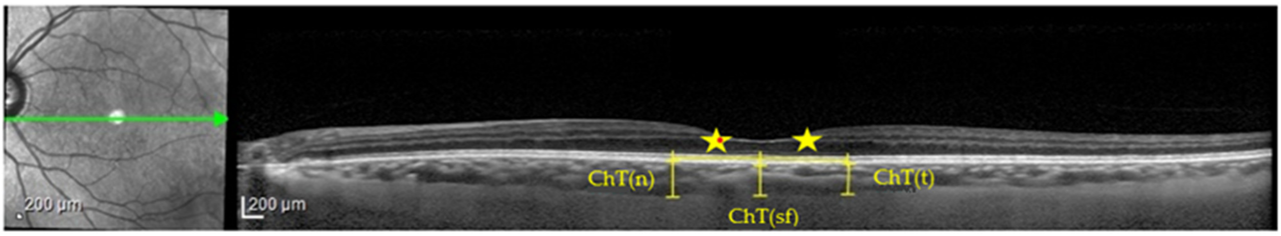

Legend: ChT(n), nasal choroidal thickness; ChT(sf), subfoveal choroidal thickness; ChT(t), temporal choroidal thickness:★, 750 microns of horizontal distance from fovea.

Figure S2. Retinal layer's (GCL, IPL, OPL, IRL respectively) thickness measurement in 5 central macular regions: a PSP patient.

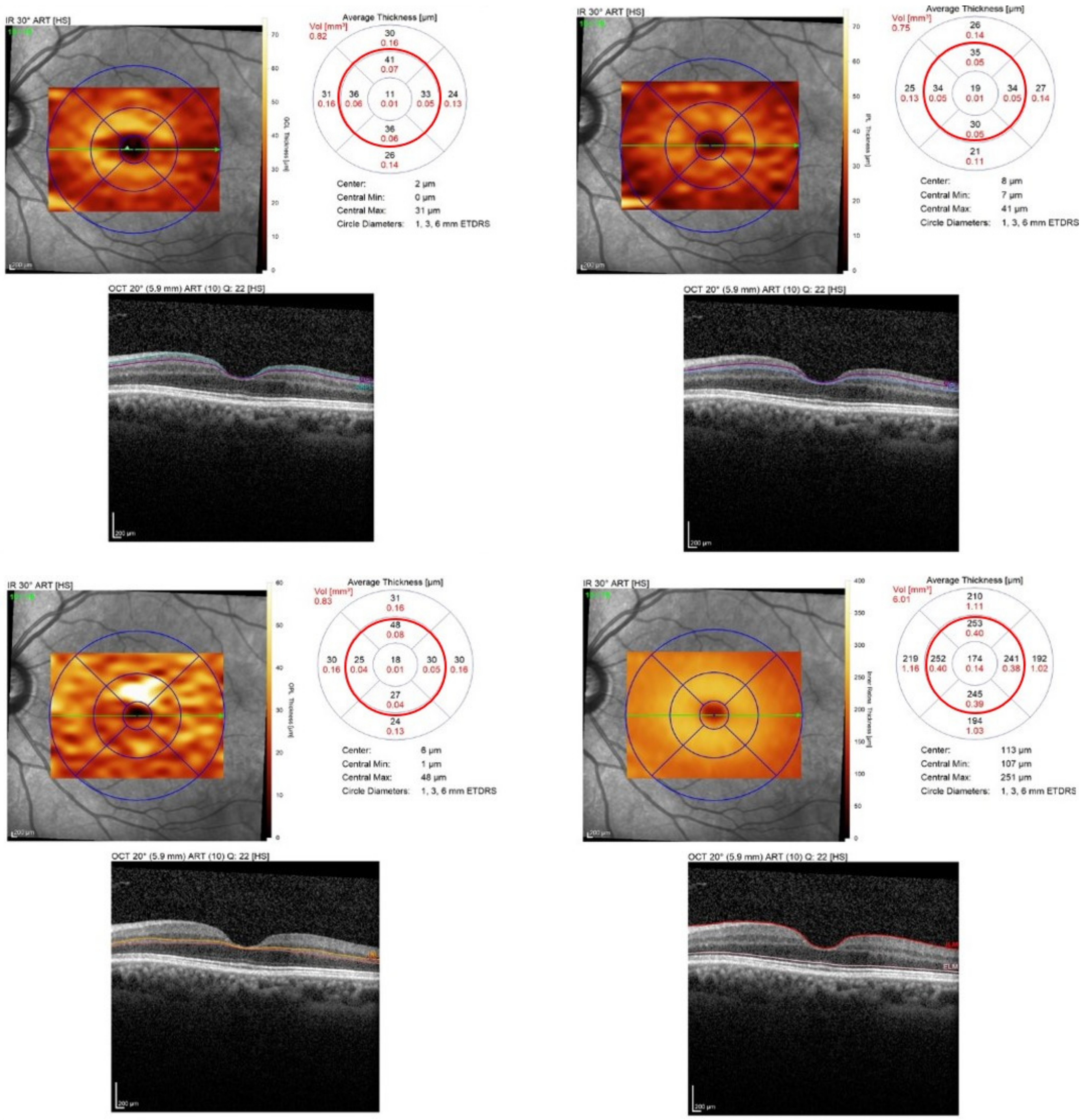

Legend: red circle, 5 central macular regions.

Abbreviations: GCL, ganglion cell layer; IPL, inner plexiform layer; OPL, outer plexiform layer; IRL, inner retinal layers.

Figure S3. Retinal layer's (GCL, IPL, OPL, IRL respectively) thickness measurement in 5 central macular regions: a HC patient.

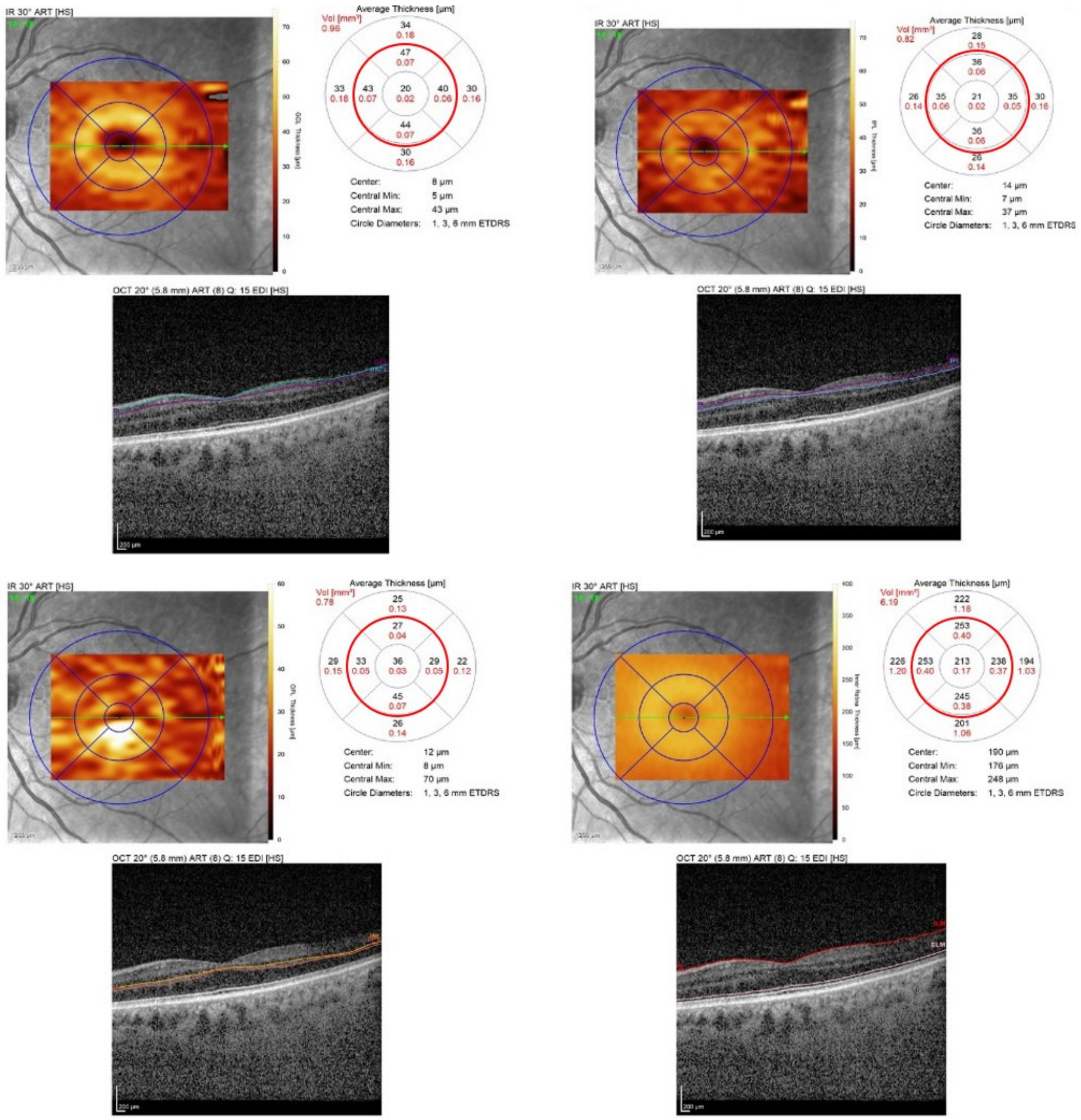

Legend: red circle, 5 central macular regions.

Abbreviations: GCL, ganglion cell layer; IPL, inner plexiform layer; OPL, outer plexiform layer; IRL, inner retinal layers.
